# Ferroptosis, necroptosis and cuproptosis: Novel forms of regulated cell death in diabetic cardiomyopathy

**DOI:** 10.3389/fcvm.2023.1135723

**Published:** 2023-03-10

**Authors:** Dan Ke, Zhen Zhang, Jieting Liu, Peijian Chen, Jialing Li, Xinhai Sun, Yanhui Chu, Luxin Li

**Affiliations:** ^1^ College of Life Sciences, Mudanjiang Medical University, Mudanjiang, China; ^2^ Heilongjiang Key Laboratory of Anti-Fibrosis Biotherapy, Mudanjiang Medical University, Mudanjiang, China; ^3^ School of First Clinical Medical College, Mudanjiang Medical University, Mudanjiang, China; ^4^ Department of Thoracic Surgery, Union Hospital, Fujian Medical University, Fuzhou, China

**Keywords:** diabetic cardiomyopathy, ferroptosis, necroptosis, cuproptosis, regulatory cell death

## Abstract

Diabetes is a common chronic metabolic disease, and its incidence continues to increase year after year. Diabetic patients mainly die from various complications, with the most common being diabetic cardiomyopathy. However, the detection rate of diabetic cardiomyopathy is low in clinical practice, and targeted treatment is lacking. Recently, a large number of studies have confirmed that myocardial cell death in diabetic cardiomyopathy involves pyroptosis, apoptosis, necrosis, ferroptosis, necroptosis, cuproptosis, cellular burial, and other processes. Most importantly, numerous animal studies have shown that the onset and progression of diabetic cardiomyopathy can be mitigated by inhibiting these regulatory cell death processes, such as by utilizing inhibitors, chelators, or genetic manipulation. Therefore, we review the role of ferroptosis, necroptosis, and cuproptosis, three novel forms of cell death in diabetic cardiomyopathy, searching for possible targets, and analyzing the corresponding therapeutic approaches to these targets.

## 1. Introduction

In 2021, worldwide, the number of adults with diabetes reached 537 million (10.5%), with more than one-in-ten adults affected by the disease. The number of people in 2021 is 7.4 million more than that in 2019, an increase of 16%, which highlights the prevalence of diabetes worldwide. The international diabetes federation (IDF) estimates that this number will reach 783 million by 2045, a 46% increase that is more than double the projected population growth of 20% over the same period, with one-in-eight adults likely to have the disease (1).

Diabetic cardiomyopathy (DCM), a disease common to most diabetic patients, is based on changes in the cardiac structure and systolic and diastolic functions. When patients have cardiac insufficiency, other clear causes, such as hypertension and structural or ischemic heart disease can be diagnosed (2). However, due to limits in awareness and a lack of diagnostic means, the current clinical diagnosis rate of diabetic cardiomyopathy is low, and the corresponding targeted clinical intervention is insufficient.

In general, the intervention involves conventional sugar control measures such as diet and hypoglycemic drugs, in the early stage. When the disease progresses to the stage of heart failure, the corresponding treatment is given according to the current guidelines for heart failure (3). However, the pathogenesis of diabetic cardiomyopathy is different from that of ordinary cardiomyopathy, which involves various factors, such as abnormal glucose and lipid metabolism, calcium balance disorder, etc. Therefore, treatment by ordinary means may lead to a poor prognosis (4).

In fact, the major risk factors for the development of diabetic cardiomyopathy are the progressive death of cardiomyocytes, changes in endothelial cells (5), and cardiomyocytes (6) in the high glucose environment. As early as 15 years ago, it was confirmed that myocardial apoptosis in patients with diabetic cardiomyopathy was 85 times more than that in the control group based on clinical biopsies (7). As a recognized form of cell death in diabetic cardiomyopathy, apoptosis has been studied extensively and intensively. However, the pathophysiological and clinical significance of this mode of cell death is inconclusive. Therefore, expanding the study of death modalities beyond apoptosis and pyroptosis will help pinpoint the target of cell death to alleviate or even cure the onset of diabetic cardiomyopathy. Recently, there have been numerous studies on regulatory cell death. More forms of death have been discovered, such as pyroptosis, immunogenic cell death, ferroptosis, necroptosis, cuproptosis, exocytosis, etc. These various forms of cell death have been proven to play a role in many diseases. From these studies, we will mainly discuss ferroptosis, necroptosis, and cuproptosis, three relatively newly identified forms of cell death. We will search for possible targets of these three forms in diabetic cardiomyopathy and analyze the corresponding therapeutic approaches according to these targets.

## 2. Ferroptosis, necroptosis, and cuproptosis in DCM

Much attention has been paid to the mechanisms related to diabetes. In recent years, oxidative stress, inflammation, and Ca^2+^-related dysfunction have been identified, as well as changes in substrate metabolism, insulin signal transduction, gene regulation, mitochondrial dysfunction, endoplasmic reticulum (ER) stress, neurohumoral activation, and cardiac cell death (8, 9). Oxidative stress is the production of excessive reactive oxygen species (ROS). In addition to the increased ROS production in diabetic patients, the endogenous antioxidant mechanism of diabetic patients is often impaired (10, 11). In diabetic animal models, intervention with appropriate antioxidants, such as superoxide dismutase (SOD) mimics or coenzyme Q10, has determined the relationship between oxidative stress and diabetic cardiomyopathy, whether as an early preventive measure to limit the progression of diabetic cardiomyopathy or after the heart has been damaged (12, 13). In addition to the direct oxidative damage caused by inappropriate cardiac ROS levels, ROS is also a key trigger for the activation of inflammatory bodies (10, 14). ROS activates a variety of other pathological signaling cascades, such as the protein kinase C (PKC), apoptosis signal-regulated kinase-1 (ASK-1), p38 mitogen-activated protein kinase (p38-MAPK), NH2 terminal JUN kinase (JUNK), and JAK-STAT pathways (8, 15–18). These signals themselves are also related to diabetes-induced cardiac complications (19, 20). Some of these signals, such as PKC, p38-MAPK, and JAK-STAT, can also induce the generation of ROS, thus forming a harmful feedforward loop. Therefore, we believe that diabetic cardiomyocytes are in a high glucose microenvironment for a long time, resulting in the activation of a variety of signaling pathways, that causes cell death. Cell death is a normal cellular phenomenon. Since the ultrastructural characteristics of programmed cell suicide were determined in 1972 (21), programmed cell death with cell apoptosis as the “representative name” has gradually come into the view of scientists, and related research has been a hot spot in the field of life science. A large number of new cells are generated every day along with the death of old cells. The normal development and maintenance of the homeostasis of living organisms as well as the elimination of damaged, senescent, and infected cells all depend on a closely regulated programmed cell death. With the deepening of research, it was found that apoptosis is not the only form of cell death. According to the different mechanisms of cell death, regulated cell death can be divided into apoptosis, pyroptosis, ferroptosis, necroptosis, cuproptosis, etc. The purpose of this review is to describe the frontier research of ferroptosis, necroptosis, and cuproptosis in DCM, to sort out the research results and current situation, to point out areas for further exploration and possible research directions, and to provide some reference for future research. Ferroptosis, necroptosis, and cuproptosis differ from other forms of cell death based on morphology, biochemistry and immune status (Table 1).

**Table 1:** Comparison of ferroptosis, necroptosis and cuproptosis.

	Ferroptosis	Necroptosis	Cuproptosis
**Definition**	Iron dependent Different from apoptosis, necrosis and pyroptosis Novel ways of programmed cell death	The death of local tissue cells *in vivo* characterized by changes in enzymatic solubility	Depending on the accumulation of copper in the cell, copper directly bind to the fatty acylated components in the TCA cycle, resulting in the aggregation and imbalance of these proteins, blocking the TCA cycle, triggering protein toxic stress and inducing cell death
**Nucleus**	Not much has changed	Perinuclear spatial focal expansion	Not much has changed
**Chromatin**	No chromatin concentration	Condensation of chromatin	No chromatin concentration
**Morphological characteristics**	Mitochondrial volume decreased, the density of bilayer membrane increased, mitochondrial crest decreased or disappeared, and mitochondrial outer membrane ruptured	Cells and organelles swell, chromatin moderately concentrates, cell membranes rupture, and cell components overflow	No morphological features
**Biochemical characteristics**	Iron accumulation Lipid peroxidation	ATP levels go down RIP1, RIP3 and MLKL are activated	Copper accumulation
**Immune characteristics**	Proinflammatory	Proinflammatory	Proinflammatory
**Cellular action**	Regulates tumor cell growth and cell death	Virus defense mechanism	Cell death targeting the TCA cycle
**Precipitating factors**	Iron accumulation	Caspase8 inhibited	Copper accumulation
**Key indicators**	Fe, GSH, MDA, GPX4, ROS, LPO	RIPK1, RIPK3, MLKL	Cu, FDX1, DLAT, LIAS, Pyruvate acid, 2-Ketoglutaricacid, HSP70
**Reference**	(22, 23)	(24, 25)	(26, 27)

### 2.1. Ferroptosis

Ferroptosis, named in 2012, is a new cell death mode and has become a focus of recent research. It is iron-dependent regulatory cell death that is different from other programmed cell death and is characterized by lipid peroxidation and iron overload (28). Since the concept was first proposed a decade ago, we have seen an explosion of research in this field, with more than 5,000 papers on ferroptosis. As a form of regulatory death, ferroptosis is significantly different from classical regulatory cell death, such as pyroptosis, especially in metabolic heart disease (29–31). Therefore, studying the mechanism of ferroptosis may reveal targets to control the progression of DCM and reduce mortality.

Currently, it is believed that the accumulation of phospholipid high oxides (PLOOHs) directly leads to ferroptosis (22). Spontaneous lipid peroxidation is initiated by ROS, and intracellular soluble ROS, including superoxide, hydrogen peroxide, and hydroxyl radicals. Among these, hydroxyl radicals are the most active and toxic ROS (32), which can rob the hydrogen atoms of phosphorylated PUFAs and form peroxides (PUFA-PL-OOH), directly leading to the death of iron. We will describe this process by splitting and tracing.

#### 2.1.1. Mechanism

##### 2.1.1.1. Source: PUFA-PL

In human cells, Acetyl-CoA is a global currency that mediates carbon trading among the metabolic pathways, including glycolysis, the tricarboxylic acid cycle, amino acid metabolism, gluconeogenesis, and fatty acid synthesis (33). Acetyl-CoA in the mitochondria is catalyzed by ACC to produce unsaturated fatty acid PUFA. In addition, dietary habits also affect the content of free unsaturated fatty acids (34). However, PUFA cannot directly cause ferroptosis (35), which needs to be further catalyzed by ACSL family members into phosphorylated PUFA (PUFA-PL), or lipid metabolism that also produces a small amount of PUFA-PL (36). PUFA-PL then enters the cytoplasm as a prelude to ferroptosis. Starting from the initial Acetyl-CoA, energy stress activates AMPK, directly inhibits ACC, and restricts the synthesis of PUFA, thus inhibiting ferroptosis (37). Correspondingly, proteins that inhibit ACSLs can also inhibit ferroptosis, such as E-cadherin, which plays a role through the Hippo-Yap pathway (38).

##### 2.1.1.2. Poison: hydroxyl free radicals

ROS include a superoxide anion (O^2·−^), H_2_O_2_, and hydroxyl radical (HO·), which are derived from the four-electron reduction reaction of oxygen in the mitochondrial electron transport chain (39). If oxygen is reduced by only one electron in this reduction process, superoxide will be produced (40). Then, superoxide is reduced to a low-reactive substance hydrogen peroxide under the action of SOD (39). Iron bivalent in cells is an active REDOX metal, which is involved in the formation of free atomic groups and the expansion of lipid peroxidation, which is one of the most destructive effects of iron-catalyzing Fenton reaction: H_2_O_2 _+ Fe^2+^→ +•OH + OH^− ^+ Fe^3+^ producing highly toxic hydroxyl radicals (41–43). Abnormal uptake, excretion, and storage of iron can lead to increased intracellular free iron and Fenton reactions (44).

##### 2.1.1.3. Key: iron

Ferric ions transported by the Tf/TfR1 protein and required by the iron uptake pathway cannot directly participate in the Fenton reaction. Ferric ions are released from Tf and become ferric bivalent after acidification from endocytosis (45). Then, ferric bivalent enters the cytoplasm by endocytosis (46), a process mainly due to ESCRT1 on the membrane (47). Therefore, both Tf/TfR1 and ESCRT are ferroptosis-promoting proteins. The role of iron is not only to promote a Fenton reaction but also to use iron as a cofactor for some key enzymes. These include arachidonic lipoxygenase (ALOX), which triggers the formation of lipid hydroperoxides that are substrates for Fenton reactions. ALOX is also involved in the peroxidation of PUFA-PL (48), and 12-lipoxygenase is required for p53-dependent ferroptosis (49). Cytochrome P450 REDOX reductase (POR) also contributes to lipid peroxidation during ferroptosis (50), suggesting that several enzymes using iron as cofactors can promote lipid peroxidation leading to ferroptosis. Of course, iron is an essential trace element in the human body (51), and the emergence of iron does not mean the death of iron. A part of the iron bivalent will be transformed into iron trivalent and stored in ferritin, and another part of it can become a polyvesicular body containing iron (MVB) mediated through a prominin-2 (prom2) solution (52). The balance of iron homeostasis depends on the expression levels and activities of ferri-carriers, ferri-transporters, and ferri-regulatory and storage proteins (53). The sensitivity of cells to ferroptosis can be regulated by controlling the level of ferritin through iron pyroptosis and further regulating the abundance of free iron because the abundance of ferritin determines the size of the labile iron pool (54).

##### 2.1.1.4. Detoxification: GPX4、DHODH、FSP1

Similar to the regulation of iron, the presence of PUFA-PL-OOH in cells does not directly lead to ferroptosis. In fact, PUFA-PL-OOH can accumulate under the action of iron bivalent, resulting in cell death through damage to cell membranes or the production of lipid-derived electrophilic small molecules (55). This accumulation process can be inhibited by CoQ10, NADPH, and BH4 (56). In addition to the inhibitory process, there is a major antioxidant defense mechanism: GPX4. GPX4, glutathione peroxidase, is a GSH-dependent enzyme that converts reduced GSH to oxidized GSH and reduces PUFA-PL-OOH to non-toxic PUFA-PL-OH (57). Based on the dependence on GPX4, GSH depletion leads to the inactivation of GPX4, causing lipid peroxide accumulation to trigger ferroptosis. GSH is an important antioxidant in cells, so it is critical to find out where GSH comes from. System Xc- on the cell membrane is an amino acid transporter that can transport extracellular cystine into the cell and intracellular glutamate into the cell, and then the intracellular cystine is reduced to cysteine, which is the rate-limiting precursor of GSH synthesis (58). GSH then serves as a cofactor for GPX4, which acts as a lipid hydroperoxidase to reduce lipid peroxides (PUFA-OOH) to the non-toxic PUFA-OH (28, 59). Therefore, GPX4 is one of the important defense mechanisms for the cellular detoxification of lipid peroxides. Normal GPX4 activity is essential for maintaining membrane lipid homeostasis, preventing the excessive accumulation of toxic lipid peroxides and the formation of free radicals (L-OOH and L-O•), thereby reducing ferroptosis (60, 61) Acyl-CoA synthetase long-chain family member 4 (ACSL4) family is an important gene promoting ferroptosis, but the sensitivity of cancer cells to GPX4 inhibitors varies depending on the cancer type, which indicates that there may be another resistance mechanism similar to GPX4-FSP1-CoQ10-NAD(*P*)H pathway (62). Ferroptosis suppressor protein 1 (FSP1), located on human chromosome 10q22.1, mediates p53-independent apoptosis (63). FSP1 was originally called apoptosis-inducing factor mitochondrial-related gene 2 (AIFM2). It specifically mediates cell death because it is caspase-independent (64). Recently, it was revealed that Ferroptosis is mediated by ubiquinone (CoQ10) (63, 65). FSP1 is enriched in the cell membrane by myristoylation. As an NAD(*P*)H-dependent CoQ10 oxidoreductase, NAD(*P*)H can be used to reduce the oxidized ubiquinone. It is known that the reduced form of ubiquinone can capture the free radicals that mediate lipid peroxidation and limit the occurrence of lipid peroxidation. Thus, FSP1 can inhibit the occurrence of ferroptosis (62). All in all, the FSP1-CoQ10-NAD(*P*) H axis may play an important role in the occurrence of ferroptosis caused by lipid peroxidation.

**Figure 1:**
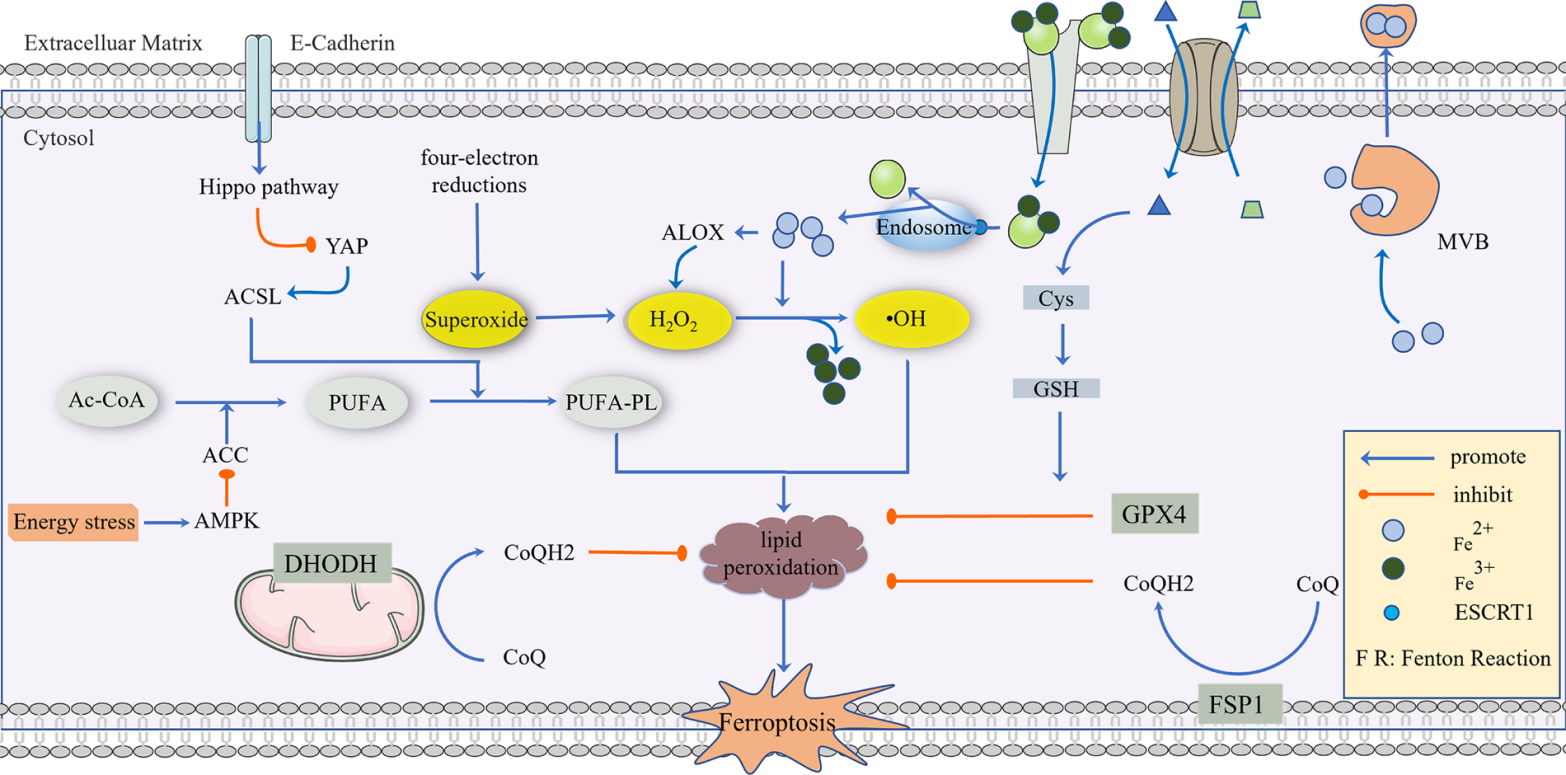
The regulatory mechanism of ferroptosis. AC-CoA in the mitochondria catalyzes the formation of PUFA under the action of ACC, which is then phosphorylated to generate PUFA-PL by the ACSL family and transferred into the cytoplasm to mediate the next stage of ferroptosis, which can be inhibited by energy stress and E-Cadherin, respectively. Superoxide produced in a mitochondrial four-electron reaction is reduced to H_2_O_2_ under the action of SOD, and a Fenton Reaction with the participation of Fe^2+^ produces highly toxic •OH, which mediates PUFA-PL peroxidation. This process can be inhibited by GPX4, CoQ10, NADPH, and BH4. GPX4 is the main antioxidant defense substance, and its inhibition of protein lipidation requires the participation of GSH. Cells transport Fe^3+^ *via* the Tf/TfR1 protein, which enters into the body through ESCRT1 on the endosome and is reduced to Fe^2+^. As a reducing agent, Fe^2+^ participates in a Fenton Reaction or as a cofactor of some key enzymes (such as ALOX), and part of Fe^2+^ can be excreted to the extracellular space *via* MVB. System Xc- can reverse the transport of Glu out of the cell membrane and Cys-Cys into the cell membrane, and Cys-Cys is further converted into Cys as a rate-limiting precursor of GSH synthesis. When excess lipid peroxides accumulate in cells, this damages to the cell membrane and eventually leads to ferroptosis.

In a study conducted by Mao C, the effect of a GPX4 inhibitor on ferroptosis in tumor cells was reversed by dihydroorotate dehydrogenase (DHODH), and this reversal was more significant in tumor cells with a low expression of GPX4, suggesting that DHODH may be the third mechanism of inhibiting ferroptosis (66). The anti-ferroptosis mechanism of DHODH is different from that of FSP1. According to research, the function of FSP1 is mainly limited to the cell membrane, and it is not clear whether it affects the mitochondrial membrane. DHODH mainly plays a role in the mitochondria. In the process of mitochondrial pyrimidine synthesis, DHODH oxidizes FMNH2 to FMN and reduces CoQ to CoQ10, thus inhibiting ferroptosis through CoQ10 capture and the scavenging of lipid peroxides (66). In addition, it has been reported that benzene-induced inflammatory anemia is associated with ferroptosis caused by the IRP1-DHODH-ALOX12 axis (67). Sorafenib is a commonly used drug in liver cancer, and its anti-tumor effect is closely related to ferroptosis. However, in recent years, the enrichment of DHODH has been found in some cells and tissues resistant to sorafenib and is related to the poor prognosis of HCC patients (68). The role of DHODH was also found in the occurrence of cervical cancer. In summary, DHODH-mediated ferroptosis in cancer is expected to become a new target for tumor therapy (66), and a nano-platform based on DHODH inhibitors has been reported to block the redox system (69) (Figure 1).

#### 2.1.2. Ferroptosis and DCM

As mentioned earlier, the system Xc-/GSH/GPX4 axis is an important pathway for ferroptosis. Many drugs function in the system Xc-/GSH/GPX4 axis, such as the classic Erastin, when ferroptosis was originally associated with this compound. Erastin inactivates GPX4 by inhibiting the system Xc- and reducing the production of GSH, promoting ferroptosis (70–72). Skipping the system Xc-, reagents that promote ferroptosis through the depletion of GSH, including acetaminophen, etc., are commonly used in clinics (73). In addition, ML162 (74) and DPI compounds (75) can also directly inhibit GPX4 and promote ferroptosis. As one of the forms of cell death, ferroptosis is involved in diabetic cardiomyopathy.

The accumulation of advanced glycation end products (AGEs) caused by hyperglycemia increases lipid peroxidation and the ferroptosis of cardiomyocytes (44), which then leads to cardiac inflammation and remodeling (cardiomyocyte hypertrophy, pro-fibrotic response and fibrosis), and ultimately leads to cardiac dysfunction, namely DCM (76). DCM inhibits the expression of SLC7A11 (system Xc- gene) and ferritin, thereby reducing GSH levels and increasing active iron levels, leading to mitochondrial oxidative damage (77). SFN plays a shielding role by activating AMPK to stimulate the downstream expression of SLC7A11 and ferritin, and at the same time, SFN enhances the AMPK dependence of the heart in age-induced ECT dysfunction and diabetic heart remodeling and dysfunction (78). These results suggest that the selective removal of excess iron inhibits ferroptosis, has a protective effect against chronic oxidative stress and that a modest reduction in plasma iron levels may be a viable treatment for the prevention of DCM in diabetic patients. From a clinical point of view, studies can test the combination of SFN with low-dose iron chelating agents, and further explore more targets of iron in the mechanism of ferroptosis depending on the effect and dose of the drug.

Increasing evidence shows that ferroptosis is involved in the onset and progression of DCM, and ferroptosis also affects the pathological consequences of cardiac function (79–82). These findings identify ferroptosis as a promising cellular target for correcting heart function in patients with DCM and possibly also for delaying the degenerative progression of DCM. However, the pathophysiological role of ferroptosis is not fully understood. Because the concept of ferroptosis is only ten years old and the data are limited, the current review does not provide any further insight into the mechanisms associated with ferroptosis and DCM, leaving many questions unanswered. For example, what role does ferroptosis play in the development of DCM, and does it play the same role at different stages in the development of DCM? In addition, all current treatments for diabetic patients are based on the premise of controlling blood sugar levels. However, the effect of controlling blood sugar levels on ferroptosis has not been determined. Whether ferroptosis affects other tissue cells in the presence of DCM has not been studied, and clearly these questions need to be further explored. Elucidating the mechanism of ferroptosis in DCM will help us develop better strategies to precisely target ferroptosis in order to effectively alleviate DCM and prevent its progression to more severe conditions. However, no such studies have been done in the literature to date, and this is a very valuable conclusion and recommendation. But up to now, no such studies have been conducted. Thus, this is a rather valuable field waiting to be explored.

### 2.2. Necroptosis

Initially, necroptosis was not considered a new form of cell death. It was morphologically similar to necrosis and shared an upstream pathway with apoptosis. Later, researchers gradually distinguished the differences between them, which led to the emergence of necroptosis (83). As a well-known mode of cell death, necrosis has long been found to be an unregulated form of cell death, caused by external physical and chemical stress, and a subsequent reaction of injury to the body (84). In contrast, necroptosis is highly regulated and serves as a defense mechanism or escape route for cells facing viral infection (85). When a viral caspase inhibitor is present, the cell can only choose to commit suicide in a way that is not caspase-dependent. The two cell death pathways, necroptosis and apoptosis, share some upstream signaling elements and ultimately lead to plasma membrane rupture. Thus, at first there was no distinction between the two forms of regulatory death, but as research progressed, it was revealed that there was a distinct difference in the cellular morphology of each process. Necroptosis is characterized by increased cell size, organelle swelling, and membrane perforation, followed by cell disintegration, the release of contents, initiation of innate and adaptive immune responses, and clearance of necrotic cells through giant cytostome (86). Meanwhile, apoptosis is characterized by cell shrinkage, cell membrane blisters, chromatin concentration, apoptotic body formation, and immunogenic protein phagocytosis through phagocytes and macrophages (87). Next, we will introduce the mechanism of necroptosis to draw out the role of necroptosis in DCM, present possible targets, and provide some suggestions for future research.

#### 2.2.1. Mechanism

Necroptosis is a unique cell death mode in vertebrate cells, which is the second line of defense established by cells to resist pathogens. When apoptosis fails, necroptosis becomes a fail-safe mechanism. Abnormal regulation of necroptosis is associated with these diseases. The expression and activity of necroptosis signaling proteins are low in cancers, such as breast cancer (88) and ovarian cancer (89), whereas the occurrence of certain inflammatory diseases, such as myocardial ischemia-reperfusion injury (90) and DCM (91), is associated with an increased expression of the signaling proteins.

**Figure 2:**
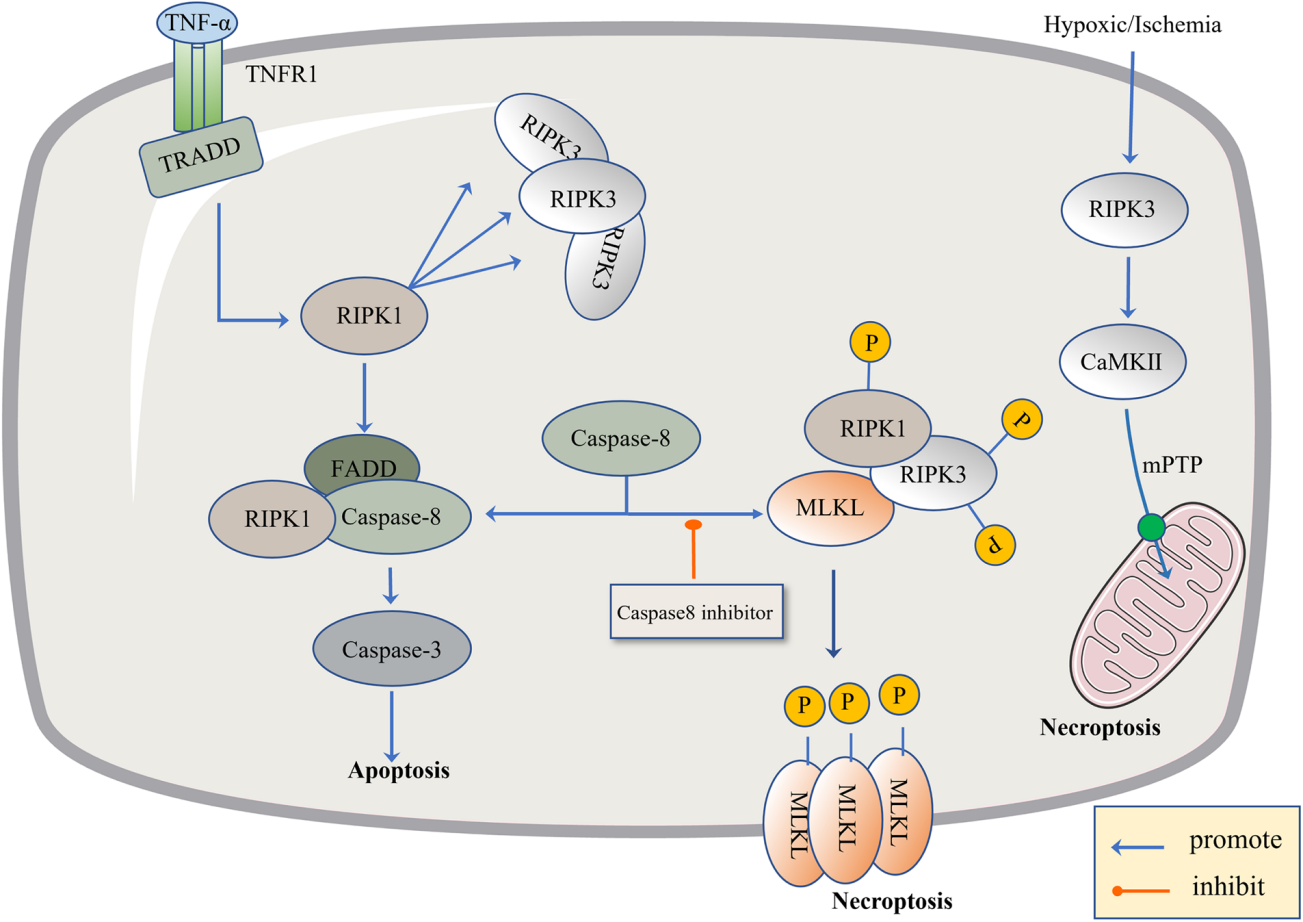
The regulatory mechanism of necroptosis. The pathogen inhibits Caspase-8, leading to apoptosis failure and the initiation of necroptosis. The level of TNF-α is up-regulated, TNF-α binds to TNFR1, activates TRADD and RIPK1, and there is the recruitment of RIPK3. When Caspase-8 is active, it promotes the combination of FADD, RIPK1 and Caspase-8, and then activates Caspase-3 to induce apoptosis. When Caspase-8 is inhibited, RIPK1 and RIPK3 are phosphorylated and bind to the phosphorylated MLKL. Phosphorylated MLKL is inserted into organelles and cell membranes, eventually leading to necroptosis. In addition, necroptosis can be activated through RIPK1-independent pathways, where RIPK3 activates CAMK II and the mitochondrial permeability transition pore (MPTP), leading to the loss of membrane potential and integrity, resulting in necroptosis.

Recently, necroptosis signaling pathways have been extensively studied. These pathways share some upstream signaling elements with apoptosis (92). Here we highlight well-studied tumor necrosis factor receptor 1 (TNFR1). The powerful TNF family has a homeostasis function (93) to defend against pathogens, in which TNF-α binds to the transmembrane protein TNFR1, allowing the TNF receptor-associated death domain (TRADD) to signal RIPK1 and recruit RIPK3 to form bad dead bodies (94). Either in terms of apoptosis or necrosis, caspase is an indissoluble protein. Thus, here, we discuss necroptosis from the caspase protein. In 2019, the journal *Nature* reported that caspase-8 induces and inhibits cell death at the same time. It induces apoptosis through death receptors such as TNFR1, and inhibits necroptosis through lysis and the inactivation of RIPK1 and RIPK3 (95). If caspase-8 is active in cells, the formation of a complex between activated caspase-8 and RIPK1 and FADD could induce apoptosis (96). If caspase-8 is inhibited, RIPK1 and RIPK3 are phosphorylated and bind to the phosphorylated MLKL, eventually leading to necroptosis (97). Caspase-8 acts like a shunt, with different gates opening and different flow directions. As a type of regulatory cell death independent of caspase, necroptosis is due to a series of automatic and cross-phosphorylation interactions between RIPK1 and RIPK3. Upon phosphorylation of RIPK3, MLKL aggregates and subsequently phosphorylates, penetrates the plasma membrane and organelles, and the membrane breakdown results in the cell contents spilling into organs, further leading to inflammatory phenotypes and damage-related DAMP releases, such as IL-1*α*, IL-*β*, and IL-33, which induce immune responses (98). DAMP sends signals to the circulatory system to recruit immune cells to damaged tissues, the component in macrophages, macro pinocytosis, and clears necrotic apoptotic cells by pinocytosis (99) (Figure 2).

#### 2.2.2. Necroptosis and DCM

The study of the role of necroptosis in DCM is relatively insufficient but still presents a high correlation. According to an animal model established by Song, using STZ-induced type 1 diabetic mice and HG-cultured rat cardiomyocytes, it was confirmed that Sirtuin 3 (SIRT3) deficiency aggravated hyperglycemia-induced mitochondrial damage, increased ROS accumulation, promoted necrosis, and possibly activated NLRP3 inflammatory. Ultimately, these processes aggravated DCM in mice (91). Recently, the correlation of the RIPK3/MLKL signaling pathway, a key pathway of necroptosis, in DCM has been confirmed. Cao et al. revealed that necroptosis of cardiomyocytes plays an essential role in mediating cardiac pathology in type 1 DCM (100). In addition, Chen et al. reduced myocardial damage, improved cardiac function, and inhibited Ca^2+^-calmodulin-dependent kinase II (CAMK II) activation through RIPK3 defects. Moreover, alleviating necroptosis in DCM mice demonstrates the existence of severe necroptosis in DCM mice (101).

It is certainly not enough to simply state a correlation. Irbesartan is a common clinical ARB drug. Existing studies have explored how irbesartan acts. Irbesartan has a cardioprotective effect on diabetic rats, and its mechanism may be related to the inhibition of the RIP1-RIP3-MLKL pathway, which alleviates necroptosis in cells. When it comes to the RIP1-RIP3 complex, AMPK inhibits the formation of this complex (102). Based on this, Cao et al. demonstrated that the anti-diabetic drug Engliazine and metformin prevented hyperglycemic-induced cardiomyopathy, which may be related to the activation of AMPK. Metformin reduces the inhibition of AMPK activation by cardiomyocytes in hyperglycemic environments (103). Therefore, the inhibition of necroptosis is a means to control DCM. More studies are needed to explore specific targets. We can consider further exploring the pathway proteins related to necroptosis, such as whether the upstream kinase mediating the regulation of caspase phosphorylation can be blocked by corresponding drugs. Do the blocking drugs have dual regulatory effects and can they regulate apoptosis at the same time? Is double regulation synergistic or antagonistic? What effect does this have on the treatment and prognosis of DCM?.

### 2.3. Cuproptosis

Similar to iron, copper is an indispensable trace element in all organisms, but it can exhibit cytotoxicity if the concentration of copper ions in cells exceeds the threshold for maintaining homeostasis (104, 105). In fact, as early as 2001, researchers found that compared with non-diabetic patients, plasma copper ion levels and urine copper ionized water are increased to varying degrees on average, while the content of copper in cardiomyocytes is decreased (106, 107). In March 2022, Peter Tsvetkov's team named a controlled cell death mode whose mechanism is clearly different from the known apoptosis by pyroptosis, necroptosis, and ferroptosis as “cuproptosis” (108).

#### 2.3.1. Mechanism

**Figure 3:**
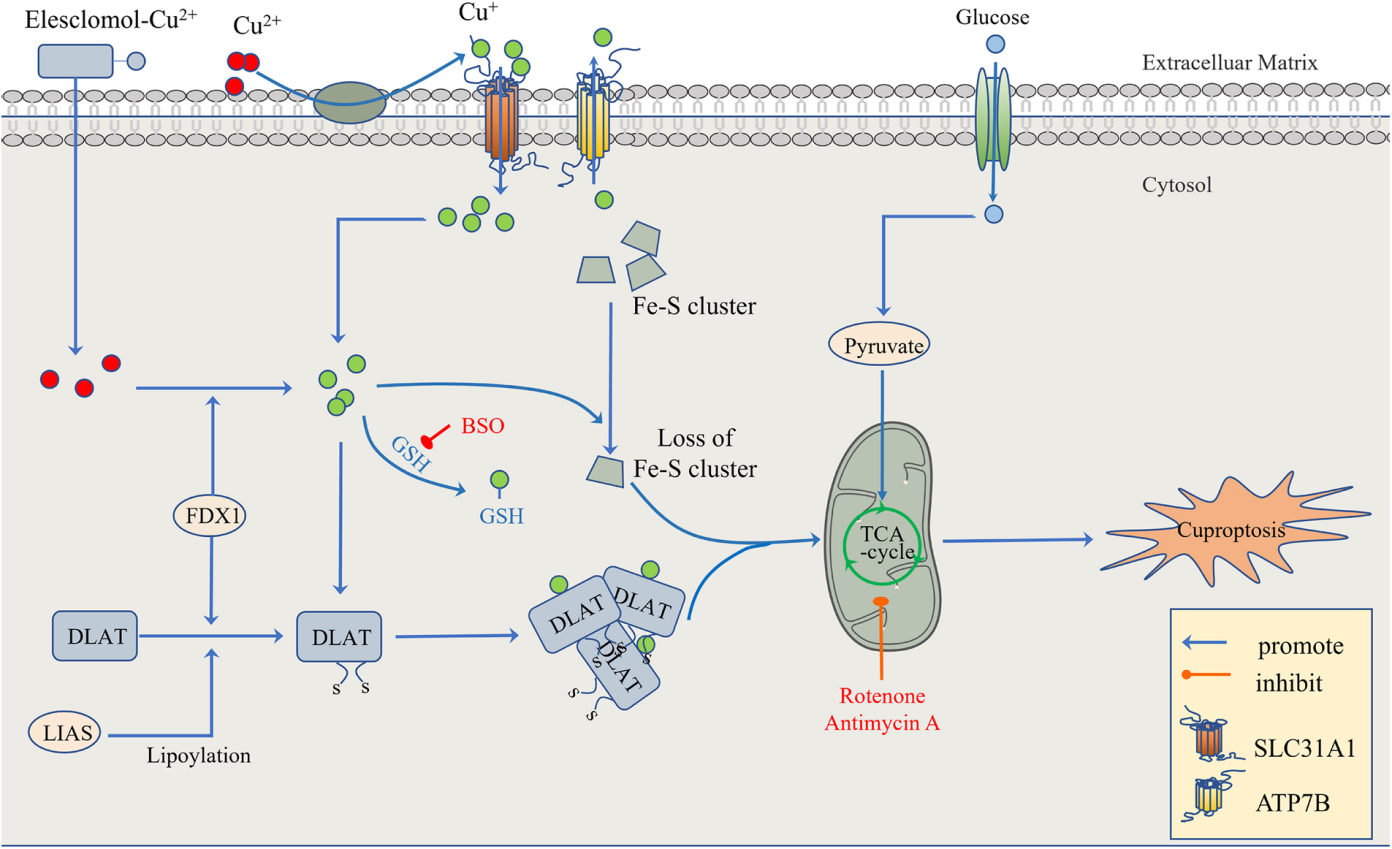
The regulatory mechanism of cuproptosis. The mitochondria are the main organelles involved in Cu-induced cell death. When the mitochondrial membrane is damaged by oxidation, the enzyme function in the TCA cycle is impaired, leading to cuproptosis. On the one hand, FDX1 and protein-lipid acylation are the key factors involved in cupric ion carrier-induced cell death. Excessive copper promotes the aggregation and functional loss of fatty acylation proteins, leading to the instability of Fe-S cluster proteins. On the other hand, FDX1 reduces Cu^2+^ to Cu ^+ ^. GSH, a thiol-containing copper chelator, blocks cuproptosis, whereas buthionine sulfoximine (BSO) promotes cuproptosis by depleting GSH. With the participation of Cu^+^, the lipid acylation and aggregation of enzymes involved in the regulation of the mitochondrial TCA cycle, such as DLAT, are promoted. Together with the instability of Fe-S cluster proteins, the mitochondrial membrane and its TCA cycle are destroyed, leading to the occurrence of cuproptosis. This process can be inhibited by electron transport chain (ETC) complex I/III inhibitors such as rotenone and antimycin A. In addition to Elesclomol-Cu^2+^, which transports Cu^2+^ into and out of cells, copper ion channels SLC31A1 and ATP7B regulate the accumulation of copper ions by mediating the entry and exocytosis of copper ions, respectively.

To confirm that this form of death is distinct from other regulatory cell death processes, researchers knocked out the key apoptosis factors BAX and BAK1 and used inhibitors of known types of cell death, including caspase inhibitors for apoptosis, and ferrostatin 1 for ferroptosis. When the cells were treated with necrostatin 1 in response to necroptosis and N-pancreatic cysteine in response to oxidative stress, the cell death induced by a copper ion carrier could not be eliminated. The data suggest that cupric ion-carrier-induced cell death is a mechanism quite different from other known modes of cell death. Interestingly, cells that rely more on mitochondrial respiration are about 1,000 times more sensitive to copper inducers than cells that rely on glycolysis. Copper deficiency damages the function of mitochondria and leads to energy reduction, thus leading to myocardial hypertrophy. A clinical investigation by Oster et al. showed that the copper ion level in cardiomyocytes was positively correlated with ejection fraction in 27 patients after coronary artery bypass grafting (109). Treatment with mitochondrial antioxidants (110), fatty acids (111) and mitochondrial function inhibitors (112) significantly changed the sensitivity of the cells to copper ions. FDX1 is the upstream regulator of protein-lipid acylation, and the loss of FDX1 leads to the complete loss of protein-lipid acylation function, as well as the accumulation of pyruvate and *α*-ketoglutaric acid and the consumption of succinic acid in cells, indicating that the loss of protein lipid acylation function blocks the progression of the tricarboxylic acid cycle (TCA) (113). FDX1 and protein-lipid acylation are key factors in the induction of cell death by copper ion carriers. Excessive copper promotes the aggregation and function loss of fatty acylation proteins, triggering the instability of Fe-S cluster proteins, leading to toxic protein stress and ultimately cell death (108) (Figure 3).

#### 2.3.2. Cuproptosis and DCM

DCM is characterized by myocardial remodeling, including myocardial fibrosis and hypertrophy. As mentioned above, cuproptosis mainly occurs in the mitochondria, which means that cuproptosis affects the course of DCM by destroying mitochondrial structure and function (78). Abnormal copper ion metabolism in cardiomyocytes is a key pathogenic process of diabetes-induced heart failure (114). Before the concept of cuproptosis was proposed, experiments were conducted to detect metabolite levels in cardiomyocytes treated with copper, and the results showed that the overall downregulation of metabolites mainly affected glycerol phospholipid metabolism, fatty acid degradation and other processes, indicating that copper can induce metabolic pathway disorder and lead to myocardial damage (115). More seriously, excessive oxidative stress is activated after copper accumulation, which disrupts the self-regulation and metabolic dynamics of the mitochondria, and the REDOX reaction cannot maintain balance, forming a vicious cycle (116).

Currently, many studies have shown that an increasing copper level can reduce myocardial hypertrophy (117–119), but for patients with DCM, the problem is not copper intake, but copper transport (120). Thus, we have taken into consideration two aspects: First, how can copper transport be regulated in patients with DCM? Is there a target for that? Second, if excessive copper is toxic after all, what is the relationship between copper toxicity and the occurrence and development of DCM? Or does copper toxicity affect the prognosis of heart failure in patients with DCM? Can copper damage be reduced by regulating plasma copper ion levels? TETA, a drug that promotes copper excretion, is currently used as an experimental treatment for diabetes to improve DCM (121) and is undergoing phase II clinical trials (122). Its principle is to promote copper excretion, prevent the excessive deposition of cardiac collagen, and improve cardiac structure and function (123). TETA has been previously approved for the treatment of Wilson disease (124), which to some extent indicates the safety of TETA.

As a newly proposed form of cell death, the current research on cuproptosis still focuses on its intervention in cancer therapy, which destroys the energy system of cancer cells by providing excessive copper to cancer tissues through targeting, leading to the death of cancer cells (108, 125), and its mechanism is still in the stage of exploration. Therefore, regarding the metabolic pathway related to cuproptosis and the influencing mechanism of its related diseases our understanding needs to be improved to find more corresponding targets. In addition, most of the current research is limited to cellular and molecular experiments, and clinical studies are rare. In the future, more studies should be carried out by combining clinical and molecular experiments.

## 3. Conclusion

**Figure 4:**
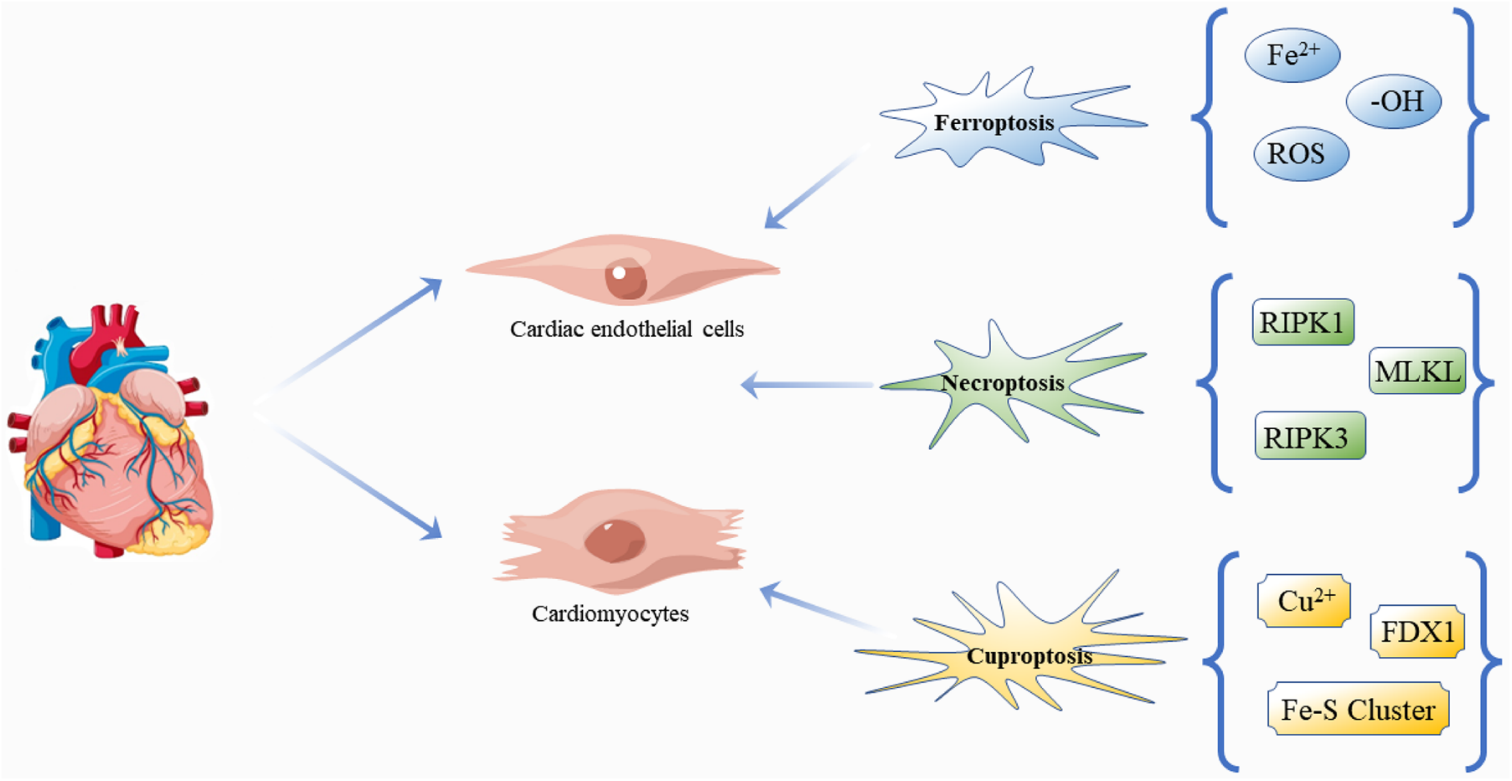
Regulatory cell death: ferroptosis, necroptosis and cuproptosis in DCM. The pathogenesis and progression of DCM are closely related to the effects of three kinds of death on cardiomyocytes and cardiac endothelial cells. Free icons were used from https://www.vecteezy.com (leaders: Shawn Rubel, Adam Gamble, Richard Fontenot).

Based on the above review, novel regulatory cell death mechanisms are involved in the cell death and progression of DCM, and it is worth noting that inhibition of any form of cell death can significantly restore damaged myocardial cell function or structure in DCM (80, 126–131), suggesting that different cell death types may be potential therapeutic targets for DCM (Figure 4). It is exciting to note that there are already regulatory cell death-related drug applications, such as Venetoclax (132), a selective Bcl-2 inhibitor in acute myelogenous leukemia, chronic lymphocytic leukemia, and small lymphocytic lymphoma. In addition, inhibiting the necroptosis of hepatocytes is an effective way to improve drug-induced liver injury. The administration of acetaminophen (300 mg/kg) and followed (1 h later) by hydroxyethidine (100 mg/kg) inhibits apoptosis and necroptosis in mice (133). Some anti-cancer drugs appear to target and enhance the ferroptosis process to kill glioma cells, including dihydroartemisinin (134) and ibuprofen (135). Inducing ferroptosis is thought to enhance the effect of traditional anti-cancer treatments that trigger other cell death pathways, such as apoptosis. However, until now, we have not thoroughly studied the mechanism of clearing these regulatory cell deaths, nor understood their pathophysiological effects. The number of studies is small, models are not perfect, and there is no reliable biomarker together with unsolved problems and limited conclusions obtained. Therefore, more studies are needed to answer the relevant questions. For example, we know that the clinical diagnosis and treatment of DM are premised on the control of blood glucose level, and of course, there is a definite relationship between hyperglycemia and DCM (136), but how hyperglycemia affects the various forms of cell death remains unclear. Do these different forms of cell death work independently? If so, what role does each play? If not, what is the connection between these different mechanisms? For example, does oxidation play the same role in each form of death at different stages of the development of DCM, and what effect does it have on the progression of the disease? Unfortunately, we have not found any studies that focus on the proportion of the various forms of death in DCM and their impact on prognosis, which may be one of the directions for further research aiming to improve the effectiveness of the treatments and survival of patients.

As mentioned above, the AMPK pathway is also an important pyroptosis pathway (137). Pyroptosis-related proteins promote mitochondrial apoptosis through ROS (138). Therefore, can one mechanism promote or inhibit other mechanisms? In addition, the associations implied by these similarities may also be targets for genetic intervention. We also noted that ferroptosis has many similarities with cuproptosis, such as the oxidative stress response caused by ROS accumulation and GSH depletion, which are related to the synergistic or antagonistic effects of drugs and may be the focus of drug development. In addition, necroptosis is more closely related to apoptosis and necrosis. The determination of the caspase protein may be a potential drug target, and the phosphorylation and subsequent modification of RIPK family may also be one direction for future drug research. In addition, there are also some similarities between death forms, such as pyroptosis, that are worth exploring.

Of particular interest to us is what these similarities represent. Is there an overlap between some forms of death? If there is overlap, are these overlapping processes an opportunity for the onset of DCM? Shedding light on these issues can help to better determine targets and target diseases. Metabolic disorders caused by diabetes are triggers for many forms of cell death. However, as mentioned above, there is currently no specific clinical treatment plan for DCM, and even treatment after heart failure only follows the guidelines for heart failure. If we can further study these forms of death, we may be able to solve this problem. After all, the incidence of DM is high and rising year by year, and cardiovascular diseases are also the main cause of death and disability at present.
